# Carbapenemases: Transforming *Acinetobacter baumannii* into a Yet More Dangerous Menace

**DOI:** 10.3390/biom10050720

**Published:** 2020-05-06

**Authors:** Maria Soledad Ramirez, Robert A. Bonomo, Marcelo E. Tolmasky

**Affiliations:** 1 Center for Applied Biotechnology Studies, Department of Biological Science, California State University Fullerton, Fullerton, CA 92831, USA; msramirez@fullerton.edu; 2 Medical Service and GRECC, Louis Stokes Cleveland Department of Veterans Affairs Medical Center, Cleveland, OH 44106, USA; robert.bonomo@va.gov; 3 Departments of Medicine, Pharmacology, Molecular Biology and Microbiology, Biochemistry, Proteomics and Bioinformatics; Case Western Reserve University School of Medicine, Cleveland, OH 44106, USA; 4 WRU-Cleveland VAMC Center for Antimicrobial Resistance and Epidemiology (Case VA CARES), Cleveland, OH 44106, USA

**Keywords:** β-lactam, β-lactamase, antibiotic resistance, plasmid, ESKAPE, *Acinetobacter*

## Abstract

*Acinetobacter baumannii* is a common cause of serious nosocomial infections. Although community-acquired infections are observed, the vast majority occur in people with preexisting comorbidities. *A. baumannii* emerged as a problematic pathogen in the 1980s when an increase in virulence, difficulty in treatment due to drug resistance, and opportunities for infection turned it into one of the most important threats to human health. Some of the clinical manifestations of *A. baumannii* nosocomial infection are pneumonia; bloodstream infections; lower respiratory tract, urinary tract, and wound infections; burn infections; skin and soft tissue infections (including necrotizing fasciitis); meningitis; osteomyelitis; and endocarditis. *A. baumannii* has an extraordinary genetic plasticity that results in a high capacity to acquire antimicrobial resistance traits. In particular, acquisition of resistance to carbapenems, which are among the antimicrobials of last resort for treatment of multidrug infections, is increasing among *A. baumannii* strains compounding the problem of nosocomial infections caused by this pathogen. It is not uncommon to find multidrug-resistant (MDR, resistance to at least three classes of antimicrobials), extensively drug-resistant (XDR, MDR plus resistance to carbapenems), and pan-drug-resistant (PDR, XDR plus resistance to polymyxins) nosocomial isolates that are hard to treat with the currently available drugs. In this article we review the acquired resistance to carbapenems by *A. baumannii*. We describe the enzymes within the OXA, NDM, VIM, IMP, and KPC groups of carbapenemases and the coding genes found in *A. baumannii* clinical isolates.

## 1. A Brief Summary of *Acinetobacter baumannii* as a Pathogen

*Acinetobacter* spp., a Gram-negative coccobacillus found in virtually all environments [1,2], used to be viewed as a “low-virulence” opportunistic pathogen of negligible significance. Despite signals about the potential this group of bacteria had as a nosocomial pathogen [3,4,5,6], its importance remained unappreciated until the mid-1990s [7]. Later, a better appreciation of the impact of *Acinetobacter* occurred after an increase in the understanding of its epidemiology identified it as the etiology of numerous hospital infections. In addition, an increase in virulence, difficulty of treatment due to drug resistance, and opportunities for infection, made this pathogen one of the most important threats to human health [8,9]. The spread and prevalence of *A. baumannii* in health care institutions was helped by its ability to withstand dry as well as humid environments, its resistance to disinfectants and antibiotics, and its biofilm-forming property that leads to colonization of inert surfaces and medical devices [10,11,12,13]. Taxonomy of the genus *Acinetobacter* has been complex, in part, due to the high genetic variability found among its members [1,11]. 

*A. baumannii,* the most common cause of nosocomial infections caused by *Acinetobacter,* is part of what is known as the *Acinetobacter calcoaceticus-baumannii* complex, a group of bacteria that also includes *Acinetobacter pittii*, *Acinetobacter nosocomialis,* and *Acinetobacter calcoaceticus* [14]. *A. baumannii* is characterized by being catalase-positive, oxidase-negative, nonfermenting, and nonpigmented (although a pigmented strain has been recently described [15]). Although it was thought to be nonmotile, that property has been disputed by newer reports [10]. *A. baumannii* infections are almost exclusively nosocomial [10,11], but community-acquired cases have been reported [10,11,16,17,18,19]. However, the vast majority of community-acquired infections occur in people with preexisting comorbidities [10,20,21]. The most common clinical manifestation of *A. baumannii* nosocomial infection is pneumonia, which has been widely reported to increase patient mortality [2,22,23]. However, some reports still dispute this fact [24]. A vast majority of these infections occur in patients undergoing mechanical ventilation in intensive care units [25]. *A. baumannii* is also responsible for bloodstream infections mainly originating from intravascular devices [10,26,27]. The mortality rates of bloodstream infections caused by this bacterium range between 30% and 52% [26,27]. Other sources of *A. baumannii* bloodstream infections are lower respiratory tract, urinary tract, and wound infections [10]. Other manifestations caused by this bacterium include, but are not limited to, burn infections, skin and soft tissue infections (including necrotizing fasciitis), meningitis, osteomyelitis, and endocarditis [28,29,30,31,32,33,34,35,36]. 

*A. baumannii* has an extraordinary capacity to attach and survive on abiotic surfaces from non-medical objects like linen or door handles to medical equipment like catheters or respirators [37,38,39,40]. This property permits this bacterium to survive in health care environments despite the highly desiccated and starvation conditions that would kill other Gram-negatives [41,42,43,44]. As a consequence, *A. baumannii* is transmitted through contact with inanimate objects, making it a constant threat to immunosuppressed and weakened patients. The ability to attach to abiotic surfaces and to resist desiccation, together with the usual multidrug and disinfectant resistance exhibited by *A. baumannii*, are the major factors behind the success of this bacterium as a nosocomial pathogen [21,45,46,47,48,49,50]. 

In the past couple of decades, *A. baumannii* was studied intensely and, as a result, some virulence factors were identified and characterized [10,20,51,52,53]. Resistance to complement-mediated killing in the vast majority of *A. baumannii* clinical isolates is due to a capsular polysaccharide, of which numerous types are identified [54,55,56,57,58]. As it is the case for other bacteria [59,60,61,62], the synthesis of the capsular polysaccharide occurs through the undecaprenol-linked glycan pathway (the Wzx/Wzy-dependent pathway) [63,64,65,66]. An additional exopolysaccharide, poly-β-(1-6)-*N*-acetylglucosamine, is biosynthesized through the synthase-dependent pathway, i.e., a single synthase protein is responsible for the polymerization and the translocation process [65,67]. This polymer seems to play a role in efficient *A. baumannii* biofilm development [68]. 

Another carbohydrate-containing macromolecule, the lipooligosaccharide, so named because the antigen-O characteristic of lipopolysaccharides is absent in this bacterium [69,70], plays a role in resistance to colistin, an antibiotic that despite its toxicity is used as a last recourse against infection [70]. Resistance occurs through modifications in the chemistry or total loss of the lipooligosaccharide lipid A [71,72,73,74]. Membrane-associated protein O-glycosylation has been associated to virulence and biofilm formation in *A. baumannii* [64,75]. O-linked glycosylation of membrane proteins shares the initial steps of the capsular polysaccharide biosynthesis [64]. The role in *A. baumannii* virulence played by pili is less clear. Evidence that type IV pili play a fundamental role in the virulence of this pathogen [70] is not available. Its synthesis has been recently associated to the presence of thioredoxin-A [76]. 

Another kind of pili, the chaperone-usher pili may play a role in biofilm formation and infection [49,70]. The Type 2 secretion systems are associated to pathogenesis or antibiotic resistance [77,78]. Information on the involvement of the Type 6 secretion system is controversial [79,80,81]. 

Another group of potential *A. baumannii* virulence factors are those systems that facilitate efficient uptake of micronutrients [82,83,84,85,86,87,88,89,90]. A paradigmatic example of these virulence factors are the iron uptake systems [91,92] that bacteria evolved in response to the host’s sequestration of iron, a group of non-specific systems of defense against bacterial infection. Despite its abundance, iron is poorly available in the host organism because it is complexed to ferritin in intracellular compartments or tightly bound to high-affinity iron-binding proteins like serum transferrin and lactoferrin [3,91,92,93,94,95,96]. To cause infection, bacteria must overcome this hurdle. Numerous iron acquisition systems were found in *A. baumannii*, and each strain possesses more than one of them. One of the systems facilitates transport of ferrous iron, known as Feo, consisting of three genes, *feoABC,* coding for a transmembrane (FeoB) and two hydrophilic cytoplasmic (FeoA and FeoC) proteins [88,97,98]. Two systems mediate the use of heme as source of iron, the *hemO* and *hemT* clusters [85,88,99,100,101]. Finally, three siderophore-mediated iron uptake systems have been identified to date. The acinetobactin system was the first to be discovered; it has been thoroughly studied, and it was shown to be critical for virulence [87,102,103]. Acinetobactin is closely related to anguibactin, another siderophore that was associated to virulence in *Vibrio anguillarum* [104,105,106]. The other two systems are those that utilize the siderophores fimsbactins (A–F) or baumannoferrins (A and B) [89,107,108]. Exhaustive and detailed descriptions of *A. baumannii* virulence factors can be found in recent excellent articles [10,20,51,53,70,78,85,109,110,111]. Other confirmed or proposed virulence factors include the phospholipases and elastase [112,113], the surface autotransporter Ata [114], the formation of outer membrane vesicles [115,116], survival factors such as serum resistance [42,117,118,119,120,121], and others [57,122,123,124,125,126]. 

Virulence of *A. baumannii* is enhanced in patients with predisposing factors such as diabetes, cancer, obstructive pulmonary disorders, immunocompromising diseases or treatment, and others [10,20,21]. Chronic alcoholism is also a risk factor for *A. baumannii* infection. Despite the rarity of community-acquired *A. baumannii* infection in the healthy population, this bacterium is a common etiologic agent of community-acquired pneumonia in individuals with a history of alcoholism [18]. Alcohol abuse causes a series of disturbances in the immune response, such as alteration of the monocytes function in presenting antigens to T-cells, alteration of the levels of cytokines and natural killer cells, as well as impairment of B-cells [127,128,129,130,131]. The detrimental effects of alcohol abuse on the immune system were shown to be a factor in the increased morbidity and mortality of *A. baumannii* [132]. 

Further studies on the alcohol-related *A. baumannii*-enhanced virulence showed elevated expression of 49 genes involved in multiple cellular functions after exposure to ethanol [125]. This study concluded that the effect of ethanol on virulence of *A. baumannii* involves numerous factors including the stress response [125]. Another study concluded that ethanol not only induces expression of certain genes, but also results in repression of others [126]. Some of the effects of ethanol described in this article include increased lipid and carbohydrate anabolism, enhanced biofilm formation, and decreased motility on semi-solid surfaces [126]. The presence of ethanol also induced the acidification of bacterial cultures and the production of indole-3-acetic acid. In summary, alcohol causes changes in the human body predisposing it for infection and induces changes that enhance or decrease expression of multiple bacterial functions that result in higher pathogenicity [125,126,132,133,134]. 

**Figure 1:**
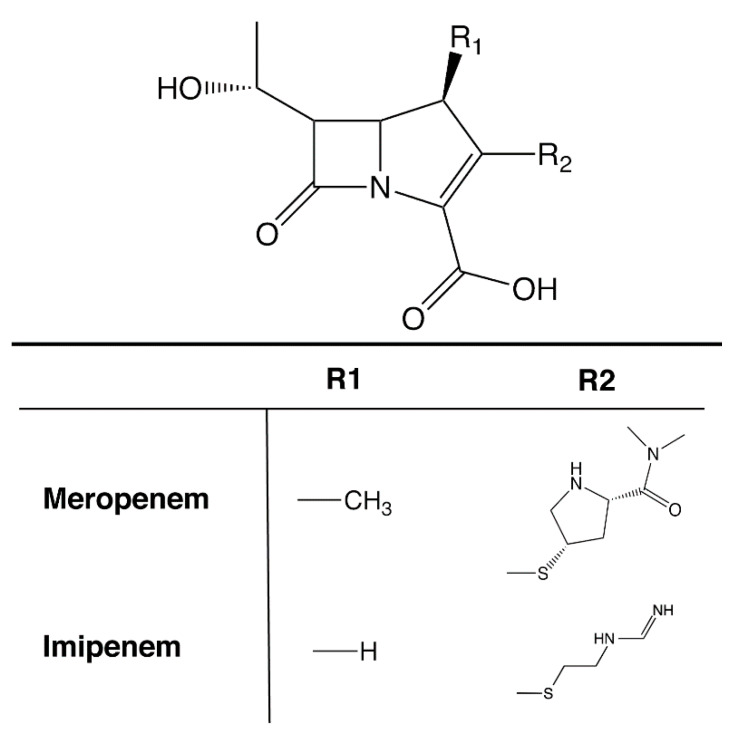
Structure of carbapenems. Generic chemical structure of carbapenems. The R1 and R2 groups for imipenem and meropenem, the most used in the clinics, are shown.

A property that turned *A. baumannii* into the human health threat that it has become is the resistance to multiple antibiotics exhibited by most clinical strains that complicate treatment [8]. In particular, resistance to carbapenems (Figure 1), which are among the antimicrobials of last resort for treatment of multidrug-resistant infections, is increasing among *A. baumannii* strains compounding the problem of nosocomial infections caused by this pathogen [9,135]. This is the reason why in the recent CDC’s 2019 Antibiotic Resistance Threats Report, Carbapenem-resistant *A. baumannii* (CRAB) is listed as an “Urgent” threat [8]. Considering the importance of this urgent threat, in this review, we focus on carbapenemase-mediated resistance to carbapenems in *A. baumannii.*

## 2. Mechanisms of Resistance to Carbapenems in *A. baumannii*

*A. baumannii* would not be the problematic pathogen it is without the multiple mechanisms of drug resistance the strains usually possess and its versatility to acquire new ones [9,51,136,137,138,139,140]. Multiple intrinsic and acquired mechanisms of resistance have been detected in *A. baumannii* isolates [51]. These mechanisms include enzymatic modification of the antibiotic, changes in permeability, efflux pumps, or modifications in the target sites [51,138,141,142,143]. Multidrug-resistant (MDR; resistance to at least three classes of antimicrobials), extensively drug-resistant (XDR; MDR plus resistance to carbapenems), and pan-drug-resistant (PDR; XDR plus resistance to polymixins) [144] *A. baumannii* strains are currently being isolated in clinics making treatment increasingly harder [9,139,145,146,147]. In particular, a “quantum leap” in the difficulty to treat *A. baumannii* infections occurred with the appearance of CRAB strains. Identified mechanisms of resistance to carbapenems include efflux pumps [51,148,149,150,151,152], reduction or inactivation of expression of porins [51,153,154,155], modification in expression or synthesis of new penicillin binding proteins [156,157], and presence of carbapenemases. Since the latter is the most frequently observed and worrisome mechanism present in CRAB strains, in this article, we review the carbapenemases detected in this bacterium. 

## 3. Carbapenemases in *A. baumannii*: OXA β-Lactamases

One of the most common, if not the most common, mechanisms of resistance to β-lactam antibiotics is their enzymatic hydrolysis mediated by β-lactamases [158,159,160]. There is more than one classification scheme for β-lactamases, which number in the hundreds, but one of the most frequently used is that proposed by Ambler (amino acid sequence-based) that divides these enzymes into four molecular classes: A, B, C, and D [160,161,162]. Enzymes belonging to classes A, C, and D catalyze the hydrolysis of the β-lactam substrate forming an intermediate covalent acyl–enzyme complex with a serine residue within the active site. In the case of class B β-lactamases, the hydrolysis reaction is mediated by one or more zinc ions [160,161]. OXA β-lactamases are class D enzymes that were originally differentiated by TEM enzymes by their ability to hydrolyze oxacillin [163]. The first OXA enzymes showed relatively narrow spectrum and were plasmid-mediated [164,165,166,167]. Later, numerous cases of proteins, encoded by chromosome- or plasmid-located genes, with characteristics of extended spectrum β-lactamase (ESBL) or carbapenemase OXA enzymes were described [160,163,168,169].

### 3.1. OXA-23-Like Group

The first OXA enzyme with carbapenemase activity to be identified in *A. baumannii* was the OXA-23 (first named ARI-1), found in an isolate from Scotland [170]. This enzyme gave the name to the first group of OXA enzymes with the capability to confer resistance to carbapenems (Table 1). Besides OXA-23, some enzymes within this group, such as OXA-27 or OXA-146, were subjected to more detailed analysis [171,172,173].

**Figure 2:**
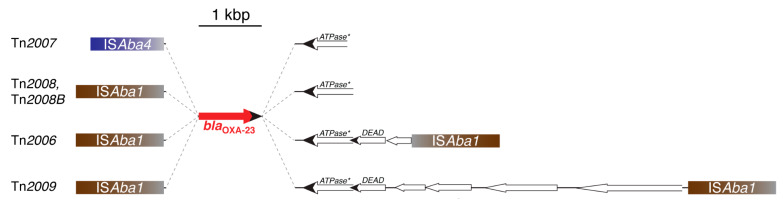
Genetic structures of *bla*_OXA-23_-containing elements. The structure of Tn*2007*, which has not been proven to transpose, is shown on top. Tn*2008* and Tn*2008B* differ in the number of nucleotides (27 and 34, respectively) that separate *bla*_OXA-23_ from IS*Aba1*. Tn*2006*, the only structure experimentally shown to transpose [178], includes copies of IS*Aba1* in opposite orientations, while Tn*2009* carries these insertion sequences in the same orientation. These two transposons are the only ones with the typical composite transposon structure. An extensive and detailed description of these elements was recently published [177]. ATPase*, gene coding for a putative ATPase truncated at the N-terminus. DADE, gene coding for a putative DEAD box family helicase [181]. Figure interpreted from Nigro and Hall (2016) [177].

The *bla*_OXA-23_ gene is thought to have originated in *A. radioresistens* where close relatives are found located within the chromosome contiguous to a gene potentially coding for an ATPase [175,176]. After the first identification, the *bla*_OXA-23_ gene was detected in *A. baumannii* isolates from several countries, usually as part of Tn*2006*, Tn*2007*, Tn*2009*, Tn*2008*, and Tn*2008B* (Figure 2). In all these elements, the *bla*_OXA-23_ is preceded by an IS element, IS*Aba1* or IS*Aba4*, and contiguous to a truncated version of the ATPase [175,177]. Tn*2006* has the typical structure of a composite transposon where the *bla*_OXA-23_ is part of a DNA stretch also containing the truncated ATPase gene and two genes, one of them coding for a helicase [177,178]. Tn*2008* and Tn*2008B* consist of a copy of IS*Aba1* associated to the fragment including *bla*_OXA-23_ and the incomplete ATPase gene (Figure 2). The difference between these two elements is the number of nucleotides between the IS*Aba1* and the OXA-23 initiation of translation codon, 27 and 34, respectively [177]. The IS*Aba1- bla*_OXA-23_*-*helicase region, i.e., the complete Tn*2008* sequence, is contained within Tn*2006*. Furthermore, Tn*2009* is a larger derivative of Tn*2006* that acquired a DNA fragment between both IS*Aba1* copies [177]. Tn*2006*, Tn*2008*, and Tn*2009* have been found within plasmids in addition to the chromosome [175,177,179,180]. Tn*2007*, detected in plasmids, consists of a DNA fragment including *bla*_OXA-23_ and a truncated version of the helicase gene preceded by a copy of IS*Ab4* (Figure 2) [177]. Table 1 summarizes the number of OXA-23-like enzymes identified to date. 

### 3.2. OXA-24/40-Like Group

The number of beta-lactamases belonging to the OXA-24/40 group is less than that of the OXA-23 group and others to be discussed below. The first representative of this group to be isolated from *A. baumannii* was OXA-24, now known as OXA-40 after sequencing revision showed that they were identical enzymes [150,182]. The number of genes in this group, species that were found, and genetic location are shown in Table 1. Some plasmid-mediated genes belonging to the *bla*_OXA24/40-like_ were found flanked by Xer site-specific recombination target sites, which led researchers to propose that this recombination mechanism plays a role in mobilization of these genes at the molecular level [183,184,185]. Xer site-specific recombination has been associated with plasmid evolution [186,187,188]. Structural studies showed that OXA-24/40 specifically utilizes carbapenems as substrate through a hydrophobic barrier for which the side chains of amino acids Y112 and M223 are essential [189]. This structure forms a tunnel-like entrance (hydrophobic bridge) to the active site. OXA-40 was also crystallized in complex to doripenem and inhibitors [190,191]. These studies were extended and details about the mechanism by which inhibitors inactivate OXA-24/40, such as decarboxylation of a K residue within, were clarified [189,191,192]. A clinical variant of OXA-24/40 identified in a clinical *A. baumannii* isolate has the mutation P227S, which results in an expanded substrate range that includes cephalosporins and aztreonam [193]. 

### 3.3. OXA-51-Like Group

OXA-51 was initially found as a chromosomally-mediated β-lactamase in a clinical *A. baumannii* isolate from Argentina [194]. Further studies showed the existence of numerous members of what is called the OXA-51-like group of enzymes [163]. The number of genes in this group, species that were found, and genetic location are shown in Table 1. Studies on the OXA-51 enzyme also showed that *bla*_OXA-51-like_ genes are specific to the *A. baumannii* chromosome. As a consequence, *bla*_OXA-51-like_ genes are used as a landmark in the identification of *Acinetobacter* isolates as *A. baumannii* [195,196]. A review describing available methodologies and their effectivity to identify *A. baumannii* has been recently published [197]. In contrast, identification of *A. baumannii* using *bla*_OXA-51-like_ as a marker can present difficulties. Although these genes are mostly located within the chromosome, a few cases of plasmid location have been detected [198]. Plasmid-mediated *bla*_OXA-51-like_ was the cause of misidentification of three non-*baumannii Acinetobacter* isolates that potentially acquired the gene from a plasmid originated in *A. baumannii* [199]. *A. baumannii* isolates from an outbreak in a hospital in Iran were hard to identify because the *bla*_OXA-51-like_ was interrupted by an insertion of IS*Aba19* [200]. 

Early biophysical and biochemical studies on OXA-51 showed that the protein did not significantly denature when exposed to broad pH (4–10) and temperature (30–60 °C) ranges, and up to 75% of the enzymatic activity was retained in these conditions [201]. Enzymatic studies showed that OXA-51-like is a weak carbapenemase and the question of whether this enzyme or others within this group are responsible for carbapenem-resistance was the focus of numerous studies [152,163,198,202,203,204]. Various *bla*_OXA51-like_ genes were found associated to IS*AbA1*, which, when in the proper orientation, enhances expression and alone or in association with expression of other mechanisms can lead to phenotypic resistance to carbapenems [204,205,206]. However, levels of expression may not be the only way OXA-51-like enzyme confers diminished susceptibility to carbapenems. Variants with one or more amino acid substitutions with respect to OXA-51 exhibit enhanced hydrolytic activity [205,207,208,209,210,211,212]. The molecular bases of several of these changes in activity associated to amino acids substitution were investigated by comparison of the three-dimensional structure of OXA-51 and the variant OXA-51 I129L in complex with doripenem [207,213]. This substitution increases the affinity of the protein for carbapenem molecules such as doripenem and imipenem. Variants including the substitution I129L show a significant increase in hydrolytic activity [208]. The results of the comparative analysis of these structures indicate that the presence of a Leu residue instead of Ile at position 129 causes the protein to better accommodate the substrate, increasing affinity and concomitantly enzymatic activity [207,213]. In conclusion, different OXA-51-like enzymes confer different degrees of or no clinical resistance to carbapenems, and they serve as a tool for molecular epidemiology of *A. baumannii*.

### 3.4. OXA-58-Like Group

This is a group of enzymes with a reduced number of variants (Table 1). The first *bla*_OXA-58_ gene was present in a non-self-transmissible 30-kbp plasmid residing in a multiresistant *A. baumannii* isolated in the Rangueil University hospital, Toulouse, France [214]. The gene was flanked by IS*Aba3*-like insertion element copies in opposite orientations. The same gene was detected in seven carbapenem-resistant isolates, six from patients, and one from the environment, in the burns unit of the same hospital [215], and rapidly spread to other countries [216]. A recent study showed that an *A. seifertii* plasmid is 99% homologous to one from *A. baumannii,* but the IS*Aba3*-like insertion element located upstream of *bla*_OXA-58_ is interrupted by a copy of IS*Aba825* [217]. These studies argue in favor of horizontal transmission as the main mechanism of dissemination of *bla*_OXA-58-like_ genes. Due to the difference in GC percent found between the gene and the *A. baumannii* chromosome, it is currently thought that *bla*_OXA-58-like_ genes originated in a different bacterium [163].

The *bla*_OXA-58_ gene was carried by plasmids harbored by six clonally related *A. baumannii* strains isolated in 2005 from a hospital in Rome, Italy. The strains showed different levels of resistance to carbapenems, and the plasmids were nearly identical but differed at the regions carrying the *bla*_OXA-58_ gene. A structure consisting of IS*26*-IS*Aba2*- *bla*_OXA-58_-IS*Aba*3 was identified that can induce amplification of the gene, producing an increase in MIC by gene dosage [218]. Gene amplification can occur through a wide variety of mechanisms [218], and its role in increasing levels of resistance has been shown multiple times [219,220,221,222,223].

### 3.5. OXA-143-Like Group 

OXA-143 was first identified in a ~30-kbp plasmid present in an *A*. *baumannii* blood culture isolate from a patient in an intensive care unit in Brazil in 2004 (Table 1) [224]. OXA-143 hydrolyzed penicillin and carbapenems but did not significantly hydrolyze expanded-spectrum cephalosporins. The *bla*_OXA-143_ gene was neither associated with insertion sequences nor located within an integron, but later, at least a *bla*_OXA-143-like_ gene, *bla*_OXA-253_, was located in a plasmid, downstream of IS*Aba47* [225]. On the other hand, *bla*_OXA-143_ and a downstream gene were flanked by copies of a gene coding for a replication protein, suggesting that its incorporation in the plasmid could have occurred by homologous recombination [224]. The OXA-143 amino acid sequence shows 88%, 63%, and 52% identity with OXA-40, OXA-23, and OXA-58, respectively. Later, other OXA-143-like enzymes were isolated in Brazil, Korea, Peru, and Honduras [225,226,227,228,229,230]. On the basis of the GC percent, these genes, which are located in plasmids, seem to have originated in a different *Acinetobacter* species [163]. 

### 3.6. OXA-235-Like Group 

The OXA-235 and two variants, OXA-236 and OXA-237, which have one amino acid substitution (OXA-236 G173V and OXA-237 D208G), were identified in nine *A. baumannii* strains from the US and Mexico [231]. Location of the genes coding for these enzymes was chromosome or plasmid, with some strains showing positive signal within a plasmid and colocalization with *bla*_OXA-51_. In all nine isolates having the *bla*_OXA-235_, the gene was flanked by two copies of IS*Aba1.* In a surveillance study performed between 2010 and 2016 in Canadian acute care hospitals, 94 carbapenem-resistant *Acinetobacte*r sp. strains, of which 90 were *A. baumannii*, were selected for further analysis [232]. OXA-235-like proteins were detected in 48% of the strains. Furthermore, IS*Aba1* was associated to the *bla*_OXA-235-like_ gene in all *A. baumannii* strains.

An outbreak that occurred across five healthcare facilities in Oregon, US, and lasted from June 2012 to October 2014, affected 16 patients and was caused by extensively drug-resistant *A. baumannii* [233]. The resistance to carbapenem was caused by the presence of the *bla*_OXA-235-like_ gene *bla*_OXA-237_, which was flanked by IS*Aba1* elements in opposite orientations in a 15.198-kbp plasmid present in all 16 isolates [233,234]. It was of special concern that *bla*_OXA-237_ is plasmid-mediated, a characteristic that enhances the potential for dissemination, and was found in a strain belonging to clonal group IC2, the most prominent worldwide [234]. 

## 4. Carbapenemases in *A. baumannii*: Metallo-β-Lactamases

The mechanism used by β-lactamases to catalyze hydrolysis of the antibiotic molecule can involve a two-step cycle consisting of acylation by formation of an acylenzyme intermediate by establishing a covalent bond between the β-lactam and a serine residue within the enzyme’s active site followed by a deacylation step or a cation-facilitated hydrolytic reaction in which one or two essential zinc ions locate at the active site of the enzyme [235]. These enzymes, known as metallo-β-lactamases or Class B β-lactamases, are subdivided into three subclasses, B1–B3 [235]. 

Enzymes belonging to subclasses B1 and B3 utilize two Zn^2+^ ions, while those within subclass B2 need just one zinc atom to catalyze inactivation. A distinctive characteristic of metallo-β-lactamases is that they are active against carbapenems [158,235,236,237]. There are still no inhibitors available for clinical use for metallo-β-lactamases. However, recently described compounds such as QPX7728 and VNRX-5133 (Taniborbactam) show inhibition activity against metallo-β-lactamases. Both compounds are active at nanomolar concentrations and could be developed as part of β-lactam/β-lactamase inhibitor formulations against metallo-β-lactamases [238,239,240].

### 4.1. NDM Group

NDM-1 is a metallo-β-lactamase (New Delhi metallo-β-lactamase) first isolated in India in 2008 from a patient with a urinary tract infection caused by a carbapenem-resistant *Klebsiella pneumoniae* [241]. The *bla*_NDM-1_ gene, located adjacent to a truncated IS*26* element, was part of a 180-kbp plasmid that includes genetic determinants that confer resistance to all antibiotics except fluoroquinolones and colistin. It was also troublesome that an *E. coli* strain isolated form the same patient harbored a plasmid containing *bla*_NDM-1_, suggesting that transfer by conjugation occurs at high frequency. This possibility was supported in mating assays and the authors predicted a worrisome scenario where the gene would spread to other bacteria [241]. Unfortunately, the predictions were correct, and bacteria harboring a plasmid containing the *bla*_NDM-1_ gene were soon isolated in infections across the world, [8,242,243,244,245,246]. 

A search at the National Center for Biotechnology Information Pathogen Detection Browser (https://www.ncbi.nlm.nih.gov/pathogens) performed at the time this article was being written produced 2407 isolates carrying NDM-1. The number of NDM-1-possesing *A. baumannii* strains was 240, only surpassed by *K. pneumoniae* (1527) and *Escherichia coli*/*Shigella* (265 combined). 

**Figure 3:**
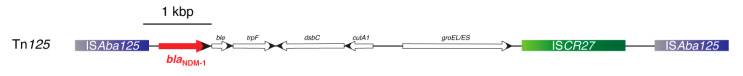
Genetic map of *A. baumannii* transposon Tn*125*. Figure interpreted from Poirel et al. [257].

Except for monobactams, NDM-1 confers resistance to all other β-lactams [247]. Twenty-seven variants were described in addition to NDM-1 that present amino acid substitutions, and in one case there is a 5-amino acid repeat. A detailed description of the NDM-1 variants has been recently published [243]. According to this publication and newer data, NDM-1, NDM-2, NDM-3, NDM-5, and NDM-7 have been detected in *A. baumannii* [243,248,249]. Unfortunately, *bla*_NDM-1_ is usually associated to other genetic determinants that specify resistance to numerous antibiotics leaving only last-line antimicrobials, usually used in combination therapies, as options for treatment [250,251,252]. While the *bla*_NDM-1_ gene was found in more than one genetic context, in all cases described to date it is located downstream of IS*Ab125* sequences that provide the -35 region of the promoter [243]. In some instances, there is complete copy of the insertion sequence and in others only a fragment (identified as ISAba125*) [253]. This structure is associated to other genes, in many cases flanked by insertion sequences to form a composite transposon [243]. In most, but not all, cases, downstream of *bla*_NDM-1_, there is a *ble*_MBL_ gene (resistance to bleomycin) followed by *trpF* (phosphoribosylanthranilate isomerase), and *dsbC* (tat twin-arginine translocation pathway signal sequence domain protein). Other genes that usually follow these genes are *cutA1* (periplasmic divalent cation tolerance protein) and *groES-groEL* (chaperonin) [243,254,255]. In many instances, this structure is followed by a copy of IS*CR27* like in the *A. baumannii* transposon Tn*125* and other elements (Figure 3) [256,257,258,259]. These structures are found flanked by different elements that imply the existence of a wide variety of mechanisms of dissemination [243,253,255,260,261,262,263]. In those cases where the structure is part of a composite transposon, this cluster of genes is flanked by IS*Aba125* (forming Tn*125*) [257], IS*903*, IS*26*, or IS*3000*, in direct or opposite orientations [243,252,253,256,264]. The structure ISAba125*-*bla*_NDM-1_-*ble*_MBL_-*trpF*-*dsbC*-*cutA1*-*groES-groEL* has been also found flanked by short repeats with features characteristic of miniature inverted repeat elements [254]. 

As it is the case with other resistance proteins like the pJHCMW1-coded AAC(6′)-Ib, which has a fusion at the N-terminus where the first six amino acids are identical to those of the TEM β-lactamase [141,142,166], analysis of the nucleotide sequence of *bla*_NDM-1_ and the upstream region showed that the first 6 amino acids of the protein are identical to those of the aminoglycoside phosphotransferase APH(3′)-Via [256], a protein associated to *A. baumannii* strains [265]. Furthermore, the GC content of the gene changes abruptly at the point of divergence. These facts, together with the known association of IS*Aba125* to *A. baumannii*, led to propose that *bla*_NDM-1_ originated through a fusion that took place in this bacterium [256]. With a few exceptions in which *bla*_NDM-1_ was found located within the chromosome, the gene is harbored in plasmids. Upwards of 350 different plasmids and 20 replicons harboring *bla*_NDM_ genes were described [243]. The rich number of plasmid varieties and transposable elements that carry *bla*_NDM_ genes explain their fast dissemination at the molecular and cellular levels [243]. The structure and biochemistry of NDM-1 has been thoroughly studied [252,266,267,268,269,270,271]. 

An interesting feature of NDM-1 is related to its dependence on zinc ions, a property shared with other metallo-β-lactamases. It is well known that one of the mammals’ innate immune responses to bacterial infections is the chelation of metal ions by high-affinity binding proteins [272,273]. Calprotectin, an important component of the cytosolic protein pool, was first identified by its ability to interfere with the growth of fungal and bacterial pathogens [274]. This property seems to be related to its ability to bind Zn^2+^ with high affinity. Calprotectin is released at the infection foci in the body, and one of its effects is to reduce the action of metallo-β-lactamases and induce degradation because the apo-enzyme is degraded in the periplasm [275]. Many metallo-β-lactamases elude this defense mechanism by being very efficient at binding the scarce zinc ions present in the periplasmic space [275]. NDM-1 differs from most metallo-β-lactamases in that limitation of zinc ions does not result in degradation because it is anchored to the outer membrane, a property that makes it refractory to destabilization [276]. Furthermore, this feature results in secretion of the protein with the release of outer membrane vesicles that shield infecting bacterial cells from high levels of β-lactams in their environment [276,277]. 

### 4.2. VIM Group 

This type of metallo-β-lactamases were first identified in *Pseudomonas aeruginosa* isolates in Italy and France [278,279]. The variants were denominated VIM-1 and VIM-2, respectively, and were part of gene cassettes as part of class 1 integrons. However, while VIM-1-carrying integron was located within the chromosome and included a second *aac(6′)-Ib*-containing gene cassette in its variable region, the VIM-2-carrying integron was part of a ~45-kbp non conjugative plasmid and possessed a unique gene cassette. The geographical location of the first detection of these enzymes and their genetic environment are represented in the VIM name of the group (Verona integron-related metallo-β-lactamase). After the first identification, the number of VIM variants increased rapidly, mainly found in *Enterobacteriaceae* [160,280,281]. Forty-six VIM variants were identified in a search at the National Center for Biotechnology Information Pathogen Detection Browser (https://www.ncbi.nlm.nih.gov/pathogens) but 70 are presently uploaded in GenBank. Early studies to identify metallo-β-lactamases in a collection of isolates from Korean hospitals showed that in one study an *A. baumannii* isolate and in the other 27 out of 38 tested *Acinetobacter* spp. samples carried VIM-2 [282,283]. Five unrelated *A. baumannii* isolates obtained in two Greek hospitals in the years 2004 and 2005 included a class 1 integron that carried *bla*_VIM-1_ in the variable portion [284]. The nontransferable characteristic of *bla*_VIM-1_ in these isolates suggests chromosomal or a nonconjugative plasmid. The gene was also detected in eight out of 13 strains tested in a study in Saudi Arabia and Iran where *bla*_VIM-2_ was also present [285,286,287]. Both *bla*_VIM-1_ and *bla*_VIM-2_ were also detected in a study of 100 *A. baumannii* isolates from three teaching hospitals in Iran [288]. Other VIM-type enzymes identified in *A. baumannii* were VIM-3 and VIM-11 in strains from a university hospital in Taiwan [289]. Several studies identified unspecified VIM-type enzymes in *A. baumannii* clinical isolates [290,291,292,293,294,295,296]. Blast analysis, carried out at the time of writing of this article, demonstrated that the genes coding for VIM-6, VIM-11, and VIM-25 were also detected in *A. baumannii*.

### 4.3. IMP Group 

**Figure 4:**
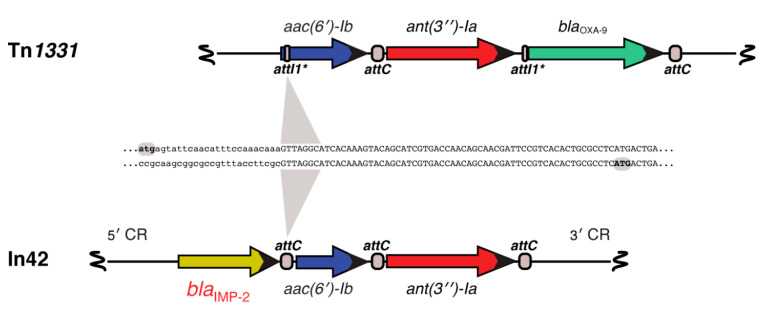
Comparison of the In42 variable region and a Tn*1331* resistance genes fragment. The genetic maps of the variable region of In42 and a resistance genes fragment of Tn*1331* are aligned. All *attC* loci are shown equally in the figure, but they are not identical at the nucleotide sequence level. The attI1* structure and functionality have been described [306]. The *aac(6′)-Ib* genes code for proteins that show differences at the N-termini, a known characteristic of this gene [141,309,310].

The first metallo-β-lactamase in the IMP (active on imipenem) group was identified in an imipenem-resistant *P. aeruginosa* clinical strain collected in 1988 in Japan [297]. The *bla*_IMP_ gene resided in a conjugative 47.7-kbp plasmid, pMS350, belonging to the P-9 incompatibility group that also conferred resistance to gentamicin and sulfonamide [297]. The *bla*I_MP_ gene was then found in the chromosome and as part of an integron in a transferable plasmid of urinary tract infection *Serratia marcescens* isolates from Japan [298,299,300]. Rapid dissemination was soon described, an expected outcome due to the location in transferable plasmids. *P. aeruginosa* and *K. pneumoniae* clinical strains carrying *bla*_IMP_ were identified, and a six-years study at the Nagasaki University Hospital, Japan, identified *bla*_IMP_ in 80 clinical strains belonging to the species *P. aeruginosa*, *P. putida*, *P. stutzeri*, and *Citrobacter freundii* [301,302,303]. The first report of an IMP-type enzyme in Europe occurred in a multidrug-resistant *A. baumannii* strain isolated from the respiratory tract of an intensive care unit patient in Italy. Further analysis showed that the gene coded for IMP-2 and was carried in a gene cassette as part of a class I integron located in the chromosome (Figure 4) [304,305]. The *bla*_IMP-2_ gene cassette was located downstream of intI1, and the variable region also included *aac(6′)-Ib* and *ant(3′′)-Ia* [304]. A Blast comparison between the *aac(6′)-Ib*- *ant(3′′)-Ia* region from this transposon and that one from the Tn*1331* [306,307,308] showed 99% homology with divergence at the point of action of IntI1 at the N-terminus coding sequences of *aac(6′)-Ib*, a well-known characteristic of this gene that exhibits heterogeneous N-termini [141,309,310,311] (Figure 4). The second identification of an IMP-type enzyme in Europe was also on a nosocomial *A. baumannii* isolate in Portugal. Analysis of this gene concluded that it was a new member, named *bla*_IMP-5_ because *bla*_IMP-3_ and *bla*_IMP-4_ had been identified earlier in Asia [312,313,314,315]. IMP-4 was later found within a class 1 integron in *A. baumannii* isolates [316] and in similar integrons in *A. pitti*, *K. pneumoniae, E. coli*, and *Enterobacter cloacae* strains. In the cases of the *E. coli* and two *E. cloacae* strains, it was confirmed that the gene resided in a plasmid [317,318]. The genetic context of *bla*_IMP-4_ present in *A. junii* was not determined [319]. To date, *A. baumannii* strains carrying *bla*_IMP-1_, *bla*_IMP-2_, *bla*_IMP-4_, *bla*_IMP-5_, *bla*_IMP-6_, *bla*_IMP-8_, *bla*_IMP-11_, *bla*_IMP-14a_, *bla*_IMP-19_, and *bla*_IMP-55_, have been reported. The *bla*_IMP-55_ was identified in a study of 65 multidrug-resistant *A. baumannii* strains isolated in two intensive care units in Iran which showed that 6 of them contained integrons including *bla*_IMP-55_ genes [320].

A search at the National Center for Biotechnology Information Pathogen Detection Browser (https://www.ncbi.nlm.nih.gov/pathogens) found 85 variants of IMP metallo-β-lactamases and 865 IMP-carrying isolates, the vast majority of which were *P. aeruginosa* (240 strains), *Enterobacter* (215 strains), and *K. pneumoniae* (184 strains). Forty-one IMP-carrying *A. baumannii* isolates were found in this search, the most prevalent variants in this bacterium were IMP-1 (21 strains) and IMP-4 (3 strains).

## 5. Carbapenemases in *A. baumannii*: KPC β-Lactamases

Enzymes within the KPC (*K. pneumoniae* carbapenemase) group are class A serine carbapenem-hydrolyzing β-lactamases that were initially reported in 2001, when KPC-1 was identified in a *K. pneumoniae* strain collected in 1996 from a hospital in North Carolina [321]. The gene quickly disseminated among *Klebsiella* strains and other bacteria and a high percentage of multidrug-resistant isolates were found to carry a *bla*_KPC_ gene [322,323,324,325,326,327,328,329,330]. Unfortunately, bacteria carrying KPC enzymes are usually multidrug resistant, which seriously limits the antibiotic options for treatment; as a consequence, mortality among patients infected with KPC-harboring bacteria is high [328]. Interestingly, in some locations, there has been a decline in the isolates harboring these genes [325,331]. The quick and efficient dissemination of *bla*_KPC_ genes is due to their usual location within transposable elements, mainly Tn*4401* or close relatives [332] in conjugative plasmids [333,334,335,336]. Tn*4401* is also commonly inserted within other transposon or insertion sequences generating multidrug resistance transposable elements [337,338]. It is worth noting that although unusual, *bla*_KPC_ genes are found associated to non-Tn*4401* genetic environments [339,340,341]. A search at the National Center for Biotechnology Information Pathogen Detection Browser (https://www.ncbi.nlm.nih.gov/pathogens) found 47 variants of KPC and 8716 records. There are numerous reports of *A. baumannii* carrying undetermined *bla*_KPC_ genes [294,342,343]. Confirmed variants of *bla*_KPC_ found in *A. baumannii* include *bla*_KPC-2_ and *bla*_KPC-3_, first identified in a study to characterize the resistance to carbapenem in *A. baumannii* (CRAB) isolates from patients with burn injury in Brazil [344]. Moreover, a study of *Acinetobacter* isolates from 17 hospitals in Puerto Rico identified *bla*_KPC-4_ and *bla*_KPC-10_ [345].

Enzymes belonging to the KPC group are the only ones that recognize as substrates all FDA-approved β-lactams. KPCs are inhibited by avibactam, relebactam, and vaborbactam [160,326,346,347,348,349,350,351]. Inhibition of KPC enzymes by clavulanate, tazobactam, and sulbactam is minimal [352]. 

## 6. Final Remarks

*A. baumannii* emerged as a problematic nosocomial pathogen in the mid-1980s, when acquisition of resistance traits became significant, making treatment more challenging [11,353]. The genetic plasticity of *Acinetobacter* spp. permitted this bacterium to evolve rapidly, turning it into one the most serious threats to hospitalized patients [52,53,354,355,356]. Currently, multi- and pan-drug-resistant *A. baumannii* are ubiquitous, and options for treatment are shrinking. The rise of carbapenem-resistant *A. baumannii* strains (CRABs) further compounds the problem, which requires urgent attention to avoid expanding the number of deaths due to nosocomial infection. Among the many mechanisms causing resistance, the acquisition of carbapenemase-coding genes are the most relevant. The majority of carbapenem-resistant *A. baumannii* isolates owes this property to the presence of OXA-23. The prevalence of *bla*_OXA-23_ is, at least in part, attributed to the spread of successful global clones such us GC1 and GC2. However, the number of isolates containing NDM-1 is rapidly growing. Research efforts geared to developing new antimicrobials must be complemented with strategies to overcome the presence of these enzymes such as the introduction of new inhibitors to be used in combination with antibacterial drugs. Despite the grim perspective of witnessing the rise of strains resilient to all available treatments, investigative attempts to introduce new inhibitors and drugs give hope that options to control these infections will continue to be available.

## Figures and Tables

**Table 1:** OXA enzymes identified in *A. baumannii* *.

Enzyme Group	Genetic Location	Predominant Isolation Countries *	Isolation Source	Other Reported Species	Total Reported
OXA-23-like	Plasmid, chromosome	USA (564), India (125), South Korea (122)	Clinical (2,830) Environmental/other (1128)	*Providencia alcalifaciens, Proteus mirabilis*, *Klebsiella pneumoniae, Citrobacter freundii*, *E. coli/Shigella*, *Serratia marcescens*, *Acinetobacter* non-*baumannii, Pseudomonas aeruginosa*	4048
OXA-24/40-like		USA (100), Spain (5)	Clinical (124) Environmental/other (21)	*Acinetobacter* non-*baumannii, Klebsiella pneumoniae, Providencia rettgeri, Staphylococcus aureus*	162
OXA-51-like		Germany (8), Brazil (8), Japan (6)	Clinical (39) Environmental/other (20)	*Acinetobacter* non-*baumannii, Klebsiella pneumoniae*	88
OXA-58-like	Plasmid, Chromosome	USA (84), Spain (12), Thailand (8)	Clinical (90) Environmental/other (177)	*Providencia alcalifaciens, Klebsiella pneumoniae, E. coli/Shigella*, *Proteus mirabilis*, *Enterobacter* sp., *Acinetobacter* non-*baumannii.*	284
OXA-143-like		Brazil (3)	Clinical (3)	*Acinetobacter* non-*baumannii*	15

* The number of isolates was calculated using the US National Library of Medicine Pathogen Detection tool (https://www.ncbi.nlm.nih.gov/pathogens) and complemented using Blast [174]. Enzymes in each group have 90% identity and 96% coverage.
